# Agranulocytosis in systemic lupus erythematosus: A case report

**DOI:** 10.1002/ccr3.6511

**Published:** 2022-11-15

**Authors:** Olfa Frikha, Raida Ben Salah, Ansar Mefteh, Faten Frikha, Mouna Snoussi, Sameh Marzouk, Zouhir Bahloul

**Affiliations:** ^1^ Department of Internal Medicine Hedi Chaker Hospital Sfax Tunisia

**Keywords:** agranulocytosis, and acute pancreatitis, enteritis, pancytopenia, polyarthritis, pulmonary arterial hypertension, systemic lupus erythematosus

## Abstract

Agranulocytosis is a rare acute condition characterized by severe a < gft (neutropenia in which the neutrophils count is less than 100/mm^3^. It can be classified into two categories, inherited, and acquired. Acquired agranulocytosis is not commonly caused by auto‐immune diseases such as systemic lupus erythematosus (SLE). We report a case of a patient suffering from agranulocytosis related to SLE at disease onset, associated with other rare disease involvements.

## Abstract 2

Hematological manifestations are frequent in systemic lupus erythematosus. Agranulocytosis is a rare condition frequently caused by drug toxicity. Systemic lupus is an exceptional cause of agranulocytosis.

## 1 INTRODUCTION

Agranulocytosis is an acute condition characterized by severe and dangerous neutropenia in which the neutrophils count is less than 100/mm^3^.^1^ It is a rare condition with an estimated incidence of 6–8 cases/million people/year.^2^ It occurs in all ages and more frequently in women. It can be classified into two categories, inherited, and acquired.^1^ Acquired agranulocytosis (AA) may be caused by medications,^3^ chemicals, infections, and auto‐immune diseases. Medications are the most common etiology (about 70% of the cases).^1^ Auto‐immune diseases are less common such as systemic lupus erythematosus (SLE).

Neutropenia is a hematological manifestation of SLE. It occurs in 20%–47% of the cases.^4^, ^5^, ^6^, ^7^ It was severe and <1000/ mm^3^ in 0.8%–6% of cases.^4^, ^6^ To the best of our knowledge, no cases were reported having agranulocytosis related to SLE.

We report a case of a patient suffering from agranulocytosis related to SLE at disease onset, associated with other rare disease involvements.

## 2 CASE REPORT

This is a case of a 45‐year‐old woman, with a history of type 2 diabetes for 8 months. Diagnosed, in January 2021, with rheumatoid arthritis; she had polyarthritis affecting the two wrists, the MCPs and the PPIs, the elevation of the sedimentation, rate, and a positive rheumatoid factor at 473 IU. she was treated with diclofenac at a dosage of 150 mg/day, 10 mg/week of methotrexate (MTX), 30 mg/day of corticosteroids (CS) (0.5 mg/kg/day) for 3 weeks then gradual taper in combination with adjuvant therapy.

**FIGURE 1:**
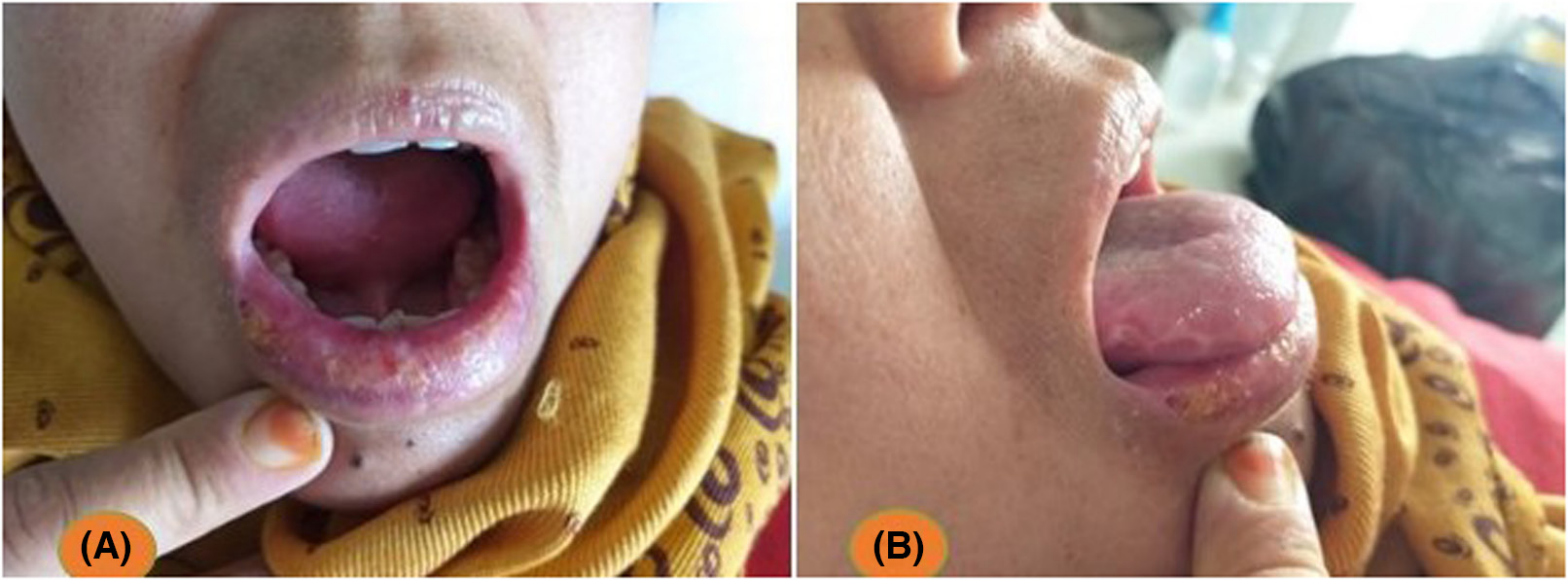
Tongue lateral border ulceration (A) and the lower lip erosions (B)

**FIGURE 2:**
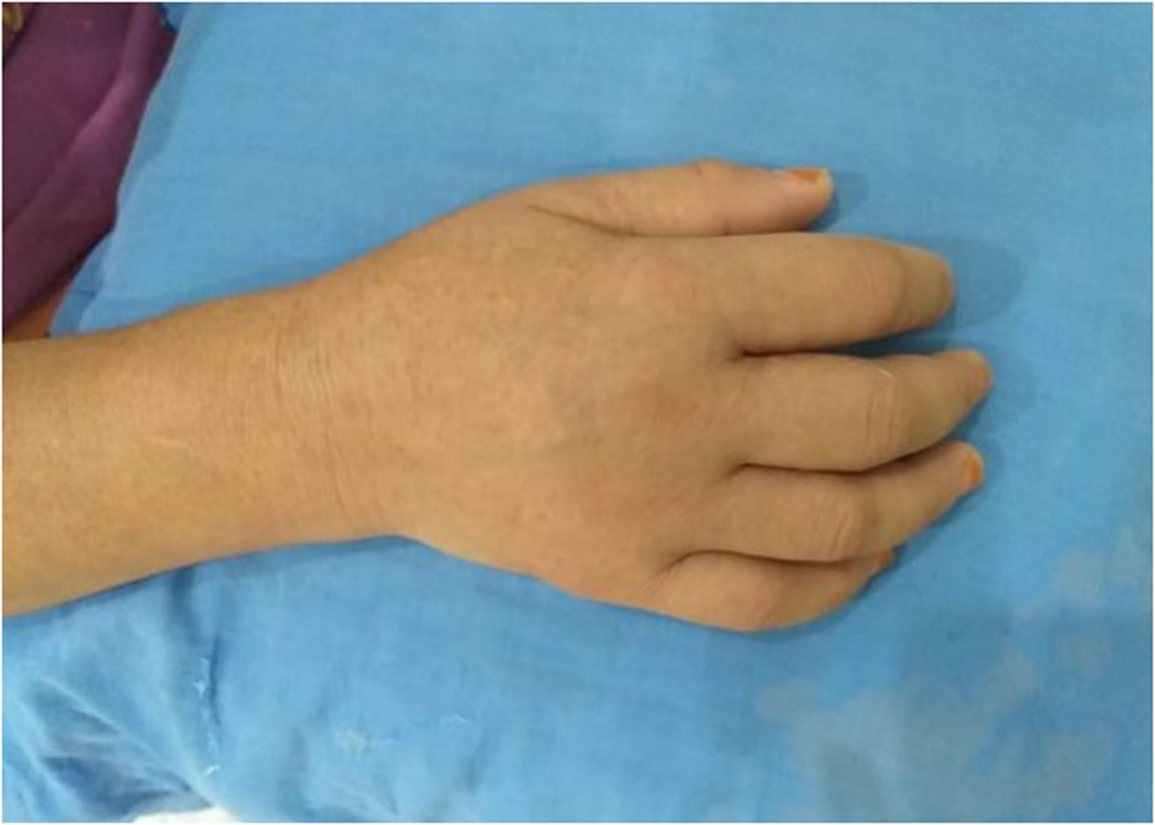
Wrist and MCP joints arthritis.

In august 2021, she was admitted to our department for a relapse of her polyarthralgia causing walking difficulties associated with neck pain and acute liquid diarrhea, she was receiving the treatment prescribed for her rheumatoid arthritis. Physical examination has shown: apyrexia, sinus tachycardia at 100 beats per minute, lower lip erosions (Figure 1A), tongue lateral border ulceration (Figure 1B), bilateral polyarthritis affecting the MCP (Figure 2), PIP and metatarsophalangeal (MTP) joints, both wrists synovitis, flessum of both elbows at 10°, left elbow arthritis, limitation of both shoulders to 90°, arthritis of both knees with flessum of 30°, and stiff cervical spine. Blood screening showed normochromic normocytic regenerative anemia at 8.9 g/dl, leukocytosis at 11170/mm^3^ with polynuclear neutrophils (PNN) predominance, lymphopenia at 420/mm^3^, biological inflammatory syndrome with a sedimentation rate >130 mm/h, CRP at 144 mg/L, and fibrinogen at 6.2 g/L. Protein electrophoresis was normal. Bacteriological examination of the urine was negative. Hands, feet, hips, pelvis, and shoulders X‐rays were normal.

The joint flare was treated with three pulses of 100 mg /day of Methylprednisolone 3 days in a row with a favorable response; the disappearance of polyarthritis and decrease of CRP level to 27 mg/L within 5 days.

Six days later, she developed febrile pancytopenia; white cells level at 610/mm^3^, hemoglobin at 7.2 g/dl with a positive direct Coombs test type immunoglobulin G, and platelets at 125,000/mm^3^. The patient was isolated and infectious investigation was performed returning negative: aerobic, anaerobic, and sabouraud blood cultures, cytobacteriological examination of urine, stool culture, parasitological examination of stool, Wright and Widal serology, serology of atypical bacteria, hepatitis B C, and HIV serology, Epstein Barr virus, cytomegalovirus, and parvoB19 virus serology. The cardiac ultrasound showed no signs of endocarditis and thoracoabdominalo‐pelvic CT showed an intraperitoneal effusion blade.

Macrophage activation syndrome was very likely due to the presence of hypertriglyceridemia at 6.1 mmoL/L, the high level of ferritin at 655.5 ng/ml and the lactate dehydrogenase level at 2.7 times normal; however, bone marrow aspiration showed no sign of hemophagocytosis but a rarefaction of all hematopoietic lines.

The albumin was decreased at 28 g/L with normal protidemia at 65 g/L. The 24‐h proteinuria fluctuated between 0.43 and 1.65 g/24 h without hematuria.

Empirical antibiotic therapy targeting a digestive infection was initiated based on cefotaxime 1 g*3/days, ciprofloxacin 500 mg/days, and metronidazole 1.5 g/days with clinical improvement in 3 days but worsening of pancytopenia; The leukopenia reached 320/mm^3^, the PNN 0/mm^3^, the lymphopenia 260/mm^3^, the thrombopenia 11,000/mm^3^, and the hemolytic autoimmune anemia (HAIA) 6.5 g/dl with an increase of CRP to 49 mg/L. Filgrastim(a human recombinant granulocyte colony‐stimulating factor [G‐CSF])at a dosage of 5 μg/kg/day and intra venous folinic acid aa t dosage of 15 mg/day were administrated for 5 days. A bone marrow aspiration showed the beginning of regeneration.

These antibiotics were replaced, 7 days later, by Tazobactam, Teicoplanin, and ciprofloxacin because of a reappearance of fever at 40°C and increase of CRP to 208 mg/L with good evolution within 2 days.

Table 1 illustrates the different laboratory features and treatment lines.

**TABLE 1:** Evolution of the laboratory features under different treatment lines

White cells (/mm^3^)	11,170	610	570	370	320	330	1200	3190	61,210	59,980	10,390
PNN (/mm^3^)	10,690	180	60	0	10	10	120	1470	38,100	29,020	4000
Lymphocytes (/mm^3^)	420	420	500	320	260	300	870	810	2850	3420	1200
Hemoglobin (g/dl)	8.9	7.2	6.8	6.5	6.4	6.7	6.7	6.3	6.1	6.6	6.5
Platelets (/mm^3^)	237,000	125,000	91,000	37,000	25,000	11,000	17,000	56,000	460,000	593,000	255,000
CRP (mg/l)	144	27	–	49	36	25	–	208	70	32	6
Creatinine (μmol/l)	334	60	61	46	46	46	–	68	–	60	65
Treatment	Pulses of methylprednisolone (100 mg/D*3D)	D1 cefotaxime, ciprofloxacin, and metronidazole		D1 Filgrastim and intravenous folinic acid	D1 CS pulses			D1 Tazobactam, D7 Teicoplanin ciprofloxacin		D13 CS	D26 CS

Abbreviations: CS, corticosteroids; D, day.

In the meantime, the immunological tests came back positive and the diagnosis of systemic lupus erythematosus was retained according to the ACR criteria with skin, joint, hematological, renal involvement, and positive anti‐nuclear antibodies (AAN) exceeding 1/1280 with elevated anti‐DNA levels (>800 IU/L) and positive anti‐nucleosome, anti‐centromere, anti‐SSAA, anti SSB, and anti RO52level.

The anti‐citrullinated cyclic peptides, antiphospholipid antibodies, cryoglobulinemia, and anti‐transglutaminase antibodies were negative.

Kidney biopsy was risky due to thrombopenia and anemia.

Pulses of methylprednisolone (1 g/day for 3 days) and hydroxychloroquine at 400 mg/day were introduced. It was associated with adjuvant therapy, iron, and folic acid. High doses of CS (1 mg/kg/day) were maintained for 8 weeks. Then, we began tapering.

Fourteen days after hospitalization, she had paroxysmal retrosternal chest pain and tachycardia at 140 beats per minute with a high level of troponin and d‐dimers and a respiratory alkalosis with a pH at 7.49. The diagnosis of pulmonary arterial hypertension was made on the cardiac ultrasound (PAH at 60 mmHg) and was attributed to SLE given the absence of pulmonary embolism on the thoracic CT angiography.

The next day, she had epigastralgia with lipasemia and amylasemia twice the normal therefore an abdominal CT was performed revealing stage B pancreatitis of Balthazar with a severity score of 4, bilateral pleuritis, and small peritoneal effusion. Triglyceridemia level was normal, as well as calcemia, the anti‐smooth muscle, anti‐liver‐kidney microsomal and anti‐mitochondrial antibodies, were negative. So, we related pancreatitis to the.

Oral nutrition was stopped for 7 days and substituted with intravenous one. Then, a semi‐liquid regimen was used.

The evolution was good with the disappearance of epigastralgia, fever, diarrhea, leukoneutropenia, and thrombocytopenia. The HAIA took time to improve. Their hemoglobin level was 6.6 g/dl. The CRP level decreased to 32 mg/L. The amylase and lipase levels were back to normal. The pericarditis and PAH disappeared on the 10th day of CS.

## 3 DISCUSSION

Agranulocytosis is usually caused by drug toxicity,^3^ infections, bone marrow diseases such as myelodysplastic syndromes and leukemias, and auto‐immune diseases such as SLE, rheumatoid arthritis, and Sjogren syndrome.^1^, ^3^ Hematological manifestations are frequent in patients with SLE.^8^, ^9^, ^10^ HAIA, leucopenia, and thrombocytopenia are common. They are mainly due to peripheral blood cell destruction by circulating antibodies or immune complex‐mediated attack or immunosuppressive therapy.^11^, ^12^ Agranulocytosis is exceptional and in majority of cases caused by drug toxicity. Unlike our patient, AA it is related to SLE.

AA physiopathology is explained by two main mechanisms^1^: inadequate or ineffective granulopoiesis, due to generalized marrow failure, occurring in aplastic anemia, leukemias, and myelodysplastic syndromes, and accelerated removal or destruction of neutrophils, due to auto‐immune reaction or is idiopathic. Its diagnosis is based on a complete blood count. Once diagnosed with AA, the patient should be isolated in a hospital room and receive systematic antibiotics with large spectrum, after doing a complete infection investigation. Every suspicious medication should be stopped. Granulocyte transfusions are no longer indicated. The use of recombinant G‐CSF can accelerate hematopoiesis and stimulate the antimicrobial function of PNN.^13^ It seems to be associated with shorter duration of neutropenia and a reduced infectious complications rate in patients who were asymptomatic at diagnosis.^13^, ^14^

Our patient was isolated once the diagnosis was made. She received large spectrum antibiotics and had a negative infection investigation. The MTX and NSAIDs were already stopped in the last 8 days. Intravenous G‐CSF and folinic acid were administered for 5 days. Within 5 days, the white cells reached 3190/mm^3^, and the PNN 1470/mm^3^. Polynucleosis and thrombocytosis occurred 9 days after G‐CSF therapy and disappeared after 27 days.

The AA prognosis is related to the septic state, the duration of agranulocytosis, early initiation of antibiotics, advanced age (>65 years), renal failure, and rapid discontinuation of the drug in question.^3^, ^13^ After the withdrawal of this drug, a latent phase occurs, varying from a few days to more than 3 weeks. Myelemia begins in 1–2 days. Neutrophilic polynucleosis starts in the following days (up to 50,000/L). A mild thrombocytosis is possible.^13^

Nonchemotherapy drug‐induced agranulocytosis is a rare adverse reaction that is due to immunologic or cytotoxic mechanisms.^15^ Anderson et al.^14^ reported in a systematic review of nonchemotherapy drug‐induced agranulocytosis that the median duration of drug exposure before a diagnosis of acute agranulocytosis ranged from 2 to 60 days. For almost 75% of drugs, this duration was >1 month.

Several infections could be responsible for neutropenia. Among viral infections, adenovirus infections have been frequently reported as a cause of febrile neutropenia.^17^ It was suggested that agranulocytosis caused by a nonimmunologic mechanism may have a later onset than the one caused by an immune‐mediated mechanism Anderson et al.^15^ also found that 67% of bone marrow examinations revealed signs of decreased generation of neutrophil granulocytes. This indicates an important difference in the pathogenesis of acute agranulocytosis and other blood dyscrasias in which peripheral cells are the main target of immune reaction.^15^ In this systematic review, they identified a list of drugs with definite or probable causality relationship with agranulocytosis with level 1 evidence (Table 2).

**TABLE 2:** Drugs with definite or probable causality relationship with agranulocytosis^15^

Category	Drug
Analgesics and NSAID	Aminopyrine, diclofenac, diflunisal, dipyrone, ibuprofen
Antiarrhythmics	Disopyramide, procainamide, quinidine
Anti‐infection drugs	Ampicillin, carbenicillin, cefotaxime, cefuroxime, flucytosine, fusidic acid, imipenem–cilastatin, nafcillin, oxacillin, penicillin G, quinine, ticarcillin
Anticonvulsants	Phenytoin
Antineoplastics	Amygdalin
Antirheumatics	Infliximab, levamisole
Antithyroid drugs	Propylthiouracil
Cardiovascular drugs	Clopidogrel, methyldopa, ramipril, spironolactone
Gastrointestinal drugs	Cimetidine, metoclopramide
Psychotropic drugs	Chlorpromazine, clozapine, fluoxetine
Other drugs	Calcium dobesilate, mebhydrolin

Treatment with hematopoietic cell growth factors seemed to be associated with a shorter duration of nonchemotherapy drug‐induced agranulocytosis and a reduction in the number of infectious and fatal complications in patients who were asymptomatic at presentation.^15^, ^18^

Methotrexate is a dihydrofolate reductase inhibitor. It can, even at a low dosage (7.5–25 mg/week), slow or halt the hematopoietic cells' maturation and reduce blood cell counts across all cell lineages.^19^, ^20^ It is eliminated mainly by the kidneys and secondarily the biliary system.^21^ Its hematological toxicity is mainly temporary. But it can be fatal in 17% of the cases.^22^

However, it was reported in the literature that pancytopenia occurs in 1.4%–2% of cases.^23^, ^24^, ^25^, ^26^, ^27^ This pancytopenia occurs precociously (<1 month) or later after 4–6 weeks of treatment beginning or doses increase.^25^ To the best of our knowledge, no AA induced by low doses of MTX was reported in the literature.

Our patient was taking MTX for 6 months. It was stopped 5 days before the pancytopenia. Although NSAID intake would have increased the hematological toxicity risk, the long duration of MTX intake (>6 weeks), its withdrawal before the pancytopenia, and the lack of AA‐induced by this drug in literature allow us to clear MTX responsibility for the AA.

So, the AA is explained, in our case, by a multisystemic flare of SLE.

Our patient presented several other particularities that make it unique and difficult to diagnose. First, she suffered from isolated polyarthritis at disease onset. Joint involvement is frequent in SLE. It is reported in 53%–76% of cases at disease onset.^27^ It has a large spectrum of manifestations going from simple arthralgia to true arthritis. They can evolve to a chronic form similar to RA in 4.7% of cases.^27^

Second, our patient presented liquid diarrhea with negative infection investigation and ascites. Although we do not have a bowl scan to confirm it, a bowl involvement remains possible. Lupus enteritis is defined as either vasculitis or inflammation of the small bowel wall that is supported by either imaging or biopsy findings.^29^, ^30^ The symptoms are nonspecific, including abdominal pain (97%), ascites (78%), nausea (49%), vomiting (42%), and diarrhea (32%).^30^ The diagnosis is based on abdominal CT findings.^29^, ^30^ CS is the first‐line treatment. The lupus enteritis generally responds well to this treatment.

Third, our patient suffered from PAH. It is defined as a mean pulmonary artery pressure (mPAP) > 20 mmHg, measured by right heart catheterization, pulmonary artery wedge pressure (PAWP) ≤ 15 mmHg, and a pulmonary vascular resistance (PVR) ≥ 3 Wood units (WU).^31^ Severe PAH is rarely seen as an initial presentation of SLE (40). Its prevalence varies between 0.5% and 17.5% in patients with SLE.^32^, ^33^ It carries a worse prognosis in SLE patients. So, prompt recognition and early treatment initiation are very important.^32^ The treatment depends on many factors including echocardiography, WHO classification functional class, exercise capacity, and hemodynamic and laboratory parameters. According to the disease severity, a combination of immunosuppressants and pulmonary vasodilators is mostly employed.^31^

Forth, our patient presented acute pancreatitis (AP). AP is a rare and severe SLE involvement.^32^ It occurs in 0.85%–9.3% of SLE patients.^34^ Wang et al.^34^ reported that SLE‐related AP frequently happens during disease activity, mostly in moderate or severe activity, and that there was multiple organ damage. For a positive diagnosis, we need to exclude other etiologies such as obstructive ones (biliary lithiasis, neoplasia, pancreas divisum), toxic‐metabolic (alcohol, hypercalcemia, and hypertriglyceridemia >11.3 mmoL/L), trauma, viral or bacterial infections, and iatrogenic (high doses of CS, immunosuppressors like azathioprine). Testing for anti‐smooth muscle, anti‐liver‐kidney microsomal, or anti‐mitochondrial antibodies should be done.^32^, ^33^, ^34^, ^35^

Systemic lupus erythematosus patients with severe pancreatitis are characterized by younger onset age, higher disease activity, fever, and multiple organ involvement (hematological, hepatic, serositis, and neurological).^33^, ^34^, ^35^ Our patient responded well to CS.

At last, we need to highlight the potential role of G‐CSF in exacerbating SLE. G‐CSF is a hematopoietic cytokine that increases neutrophil production, differentiation, and survival by stimulating the growth of neutrophil bone marrow progenitors, and the rate of maturation and release into the circulation. It also enhances adhesion and phagocytosis of mature neutrophils.^36^ It is the key cytokine that regulates neutrophils. Neutrophil dysregulation is implicated in the pathogenesis of SLE.^37^ Dramatic increases of neutrophils after G‐CSF may lead to organ damage by tissue infiltration and the release of proinflammatory cytokines.^36^ In SLE, clearance of apoptotic material may be impaired. G‐CSF administration could additionally dysregulate the inhibitory effect of neutrophil apoptosis, leading to a rapid surge of apoptotic material providing a rich source of lupus autoantigens.^38^ In the literature, several cases have experienced SLE flares associated with G‐CSF therapy.^38^, ^39^ It was reported that excessive doses of G‐CSF may increase the risk of exacerbation of SLE, particularly if the absolute neutrophil count is increased to be much higher than 1. 10^9^/L.^38^

Our patient suffered from SLE flare with skin, joint, renal, and hematological involvement before G‐CSF administration. Five days after it, she developed PAH and AP. They could be related to the initial severe flare of the disease or secondary to G‐CSF therapy. So, this therapy should be used with great caution in SLE patients.

## 4 CONCLUSION

Systemic lupus erythematosus has a large spectrum of clinical and biological manifestations. We need to keep in mind the rare involvements, to have a diagnosis and treat it on time to save the patient's prognosis.

## AUTHOR CONTRIBUTIONS

R Ben Salah and O Frikha collected data and information and were the major writers of the present work. A Mefteh and M Snoussi performed, described, and analyzed the data, and contributed to manuscript writing. Z Bahloul, F Frikha, and S Marzouk contributed to data analysis and manuscript writing.

## FUNDING INFORMATION

NONE.

## CONFLICT OF INTEREST

The authors declare that they have no competing interests.

## STATEMENT ABOUT DIGITAL PHOTOGRAPHS

The authors declare that all the digital photographs included in this work have not been modified, edited, or adulterated in any way.

## ETHICAL APPROVAL

The patient has consented to publication the case, imaging and all data.

## CONSENT

Written informed consent was obtained from the patient to publish this report in accordance with the journal's patient consent policy.

## Data Availability

Data sharing is not applicable to this article as no new data were created or analyzed in this study.
